# Clinical empathy and relational continuity mitigate care fragmentation for patients with multiple long-term conditions: a qualitative study

**DOI:** 10.3399/BJGP.2026.0171

**Published:** 2026-09-22

**Authors:** Stella Arakelyan, Bruce Guthrie, Atul Anand, Nazir Lone, Stewart W Mercer, Tricia Tooman

**Affiliations:** 1 Advanced Care Research Centre, Usher Institute, University of Edinburgh, Edinburgh, UK; 2 Centre for Cardiovascular Science, University of Edinburgh, Edinburgh, UK; 3 Royal Infirmary of Edinburgh, Edinburgh, UK

**Keywords:** clinical empathy, complex adaptive systems theory, multimorbidity, person-centred care, quality of care, primary health care

## Abstract

**Background:**

Services for patients with multiple long-term conditions (MLTC) operate within fragmented and poorly coordinated systems but how care experiences, perceptions, and behaviours emerge and adapt in response to service changes and increasing system complexity is poorly understood.

**Aim:**

To explore how experiences and perceptions of care for patients with MLTC and their carers emerge and evolve within a complex adaptive care system to identify opportunities for better care.

**Design and setting:**

Qualitative analysis of six focus groups with 28 patients with MLTC and carers in four Scottish integrated health and social care partnerships was undertaken.

**Method:**

Data were collected between February and May 2024. Abductive analysis informed by a complex adaptive systems lens was used.

**Results:**

Care for patients with MLTC emerged through dynamic, reciprocal interactions between patients, carers, professionals, and organisational structures. Service changes introduced to manage rising demand were often perceived as misaligned with patients’ complex needs, prompting patients and carers to modify their behaviours to navigate access barriers. Care experiences were unpredictable, shaped by reinforcing feedback loops in which small personal interactions had a disproportionate effect on trust and engagement. Informal care expanded to fill system gaps, with family members assuming coordination and advocacy roles. Clinical empathy and relational continuity of care acted as key stabilising influences within fragmented care systems.

**Conclusion:**

Service changes intended to improve care efficiency can inadvertently destabilise care and shift the burden onto patients with MLTC and their carers, affecting perceived care quality and relational continuity. The quality of interactions and relationships between agents shape patients’ perceptions of care and future engagement.

## How this fits in

Health and care systems are fragmented and not well suited to meet the complex and changing needs of patients with multiple long-term conditions and their carers. Exploring how experiences and perceptions of care emerge and evolve within complex care systems is important to identify opportunities for better care. Within fragmented systems, personal interactions among system agents (such as patients, carers, professionals, digital systems, and services) affect trust, perceptions of overall care, and future engagement. Informal care fills system gaps, with family members assuming care coordination and advocacy roles. Clinical empathy and relational continuity of care act as stabilising influences within fragmented systems.

## Introduction

The prevalence of multiple long-term conditions (MLTC) is rising rapidly worldwide.^1,2^ Between a quarter and a third of all adults live with MLTC,^3,4^ and prevalence is expected to rise because of an ageing population and improving survival rates, resulting in a growing population with complex, interacting, and cumulative health and care needs.^2^ Although age is a strong predictor, MLTC affect people of all ages. Because there are more middle-aged than older people, in absolute terms, more people aged <65 years live with MLTC than people aged ≥65 years. Additionally, MLTC are socially patterned, with people from more deprived communities developing MLTC 10–15 years earlier.^3^ MLTC are associated with functional decline, reduced quality of life, poorer mental health, polypharmacy, and unscheduled admissions to hospital.^2,3^

Health and care systems organised around specialist care for single conditions are particularly poorly aligned for the needs of patients with MLTC. Reforms that better account for MLTC while maintaining the benefits of specialist care are constrained by misaligned organisational priorities, poor communication, poorly integrated information systems, and limited resources.^5,6^ As a result, despite changes such as the strengthening of geriatric care and frailty pathways, care for patients with MLTC is often fragmented and poorly coordinated.^2^ Patients and carers are not passive recipients of care. They, too, develop strategies to manage MLTC and navigate fragmented care pathways, with some scholars arguing that MLTC should be framed as an experience that manifests through navigations of health and social care system infrastructures.^7^ Despite this, there is limited understanding of how patients and carers adapt and learn how to manage as complexity unfolds within health and care systems, and how these dynamic interactions with system agents (human and non-human) shape care experiences and perceived outcomes.

Complexity science, particularly complex adaptive systems (CAS) theory, provides a useful framework for exploring the multiple interacting processes that shape care for patients with MLTC and ‘what ultimately matters’ to them.^8^ CAS conceptualises health and care systems as composed of multiple interacting agents whose behaviours adapt over time, producing outcomes that are emergent rather than predictable.^9^ Although the application of complexity science in healthcare research and improvement work has been expanding,^10–12^ research that uses CAS to understand how experiences, perceptions, and behaviours of patients with MLTC and their carers emerge and adapt in response to changes and increasing system complexity remains sparse.

This study therefore aimed to explore how experiences and perceptions of care for patients with MLTC and their carers emerge and evolve within a complex adaptive care system to identify opportunities for better care. By utilising a CAS lens, we explored experiences and perceptions of care by describing interactions between key agents, the patterns of care and adaptations arising from these interactions, and the ways in which patients with MLTC and their carers co-evolved in response to system changes.

## Method

### Study design, setting, and participants

This study is part of the Artificial Intelligence and Multimorbidity: Clustering in Individuals, Space and Clinical Context (AIM-CISC) programme.^13^ The qualitative design analysed focus group discussions with a purposive sample of patients with MLTC and their carers in four health and social care partnerships (HSCPs; one city and three mixed urban and rural) responsible for integrated care delivery in a large health board serving around 17% of Scotland’s total population. HSCPs unify services previously managed separately by NHS and local authorities, providing free health care and means-tested social care.^14,15^

In collaboration with the NHS Research Scotland primary care network, we purposively sampled six general practices serving mixed urban and rural populations. Practices identified patients with MLTC and carers to participate in focus group discussions. Eligible participants were those aged ≥40 years, living with ≥2 long-term conditions, prescribed ≥7 medications, having recent experience in health and social care, and their informal carers. The age threshold of ≥40 years was chosen to capture individuals from less affluent communities who experience earlier onset of MLTC and worse outcomes. Taking ≥7 prescribed medications was used as an indicator of treatment burden and healthcare complexity, and recent experience of health and social care was defined as contact with health and/or social care services within the previous 4–6 months to minimise recall bias.

### Data collection

The research team comprised two health services researchers with backgrounds in public health and social sciences, alongside primary care researchers and clinical academics. The discussion guide was developed by two authors (the first and second authors) and pilot tested with three patients with MLTC and two carers. The guide included questions on recent experiences of health and social care, expectations and wishes in connection with suboptimal care experiences, opportunities for improving care pathways, and enhancing support services for carers (see Supplementary Box S1).

Six focus group discussions were held by a health services researcher (the first author) between February and May 2024, lasting between 95 and 120 min. Recruitment, data collection, and analysis occurred iteratively, and we continued recruiting until we reached a point of thematic saturation.^16^

### Ethical considerations

Participants were sent written information about the study by their practice and invited to contact the research team for more information if they were interested in taking part in the study. Participants returned signed consent forms before the group discussion. All participants were assured of confidentiality and anonymity and informed about their rights to withdraw from the study at any time, with any data collected up to the point of withdrawal being retained and included in the analysis.

### Analysis

Group discussions were audio-recorded, transcribed verbatim, and imported to NVivo (version 12) software for coding. Qualitative abductive analysis^17^ was used, guided by CAS theory principles of system interconnectedness, continuous adaptation, feedback loops, and resulting non-linearity in the system. The first and senior authors sensitised themselves to the dataset by reading transcripts and engaging in weekly analytical discussions over several months. The first and senior authors developed and refined conceptual categories drawing on CAS theory and used these as a coding framework (Box 1). Coding results were then discussed and further honed for clarity and consistency. To ensure transparency in analysis and reporting, we used processes allowing for an audit trail of data collection, management, and analysis.

**Box 1.** Principles of complex adaptive systems (CAS) and their descriptions

CAS principle	Description
**Diversity of agents**	Systems contain heterogeneous actors with different goals, resources, and behaviours
**Interconnectedness and relationships**	System components influence each other through dense networks of relationships
**Dynamic boundaries**	System boundaries shift as relationships and contexts change
**Non-linearity, unpredictability, and uncertainty**	Small changes can produce disproportionate and unpredictable effects
**Emergence**	System patterns emerge from local interactions rather than central control
**Feedback loops**	Interactions generate feedback that shapes system behaviour
**Self-organisation**	Agents adapt and organise themselves without external direction
**Adaptation and co-evolution**	Systems and agents continuously adjust in response to changes
**Distributed control**	No single actor controls the system, and influence is shared across many agents
**Simple rules**	Complex behaviour can arise from a few guiding principles rather than detailed instructions
**Sensitivity to initial conditions**	Starting points shape trajectories and small differences early on matter
**Path dependence**	Historical events and decisions influence current system dynamics and future trajectories

The multidisciplinary research team conducting this study has many decades of experience with patients with MLTC, as well as research interests focused on systems theories. These areas of expertise informed our perspectives, assumptions, and interpretations of findings. Findings are reported following the Standards for Reporting Qualitative Research guidance.^18^

## Results

Twenty patients with MLTC (*n* = 9 men), six carers (*n* = 5 women), and two people with MLTC sharing caregiving roles (women) participated in six focus group discussions. Over half of the participants were aged ≥70 years (mean 70 years, range 51–89 years) and were cohabiting with another person (Table 1).

**Table 1.** Participant characteristics (*N* = 28)

Characteristic	Participants, *n*
**Age, years**	
50–59	8
60–69	4
70–79	14
80–89	2
**Gender**	
Woman	18
Man	10
**Ethnicity**	
Scottish	23
Other British	5
**Region**	
Midlothian	7
East Lothian	5
Edinburgh	16
**Status**	
Patient with MLTC	20
Informal carer	6
Both	2
**Living arrangements**	
Live at home with another person	17
Live at home on their own	10
Live in sheltered (retirement) housing on their own	1

MLTC = multiple long-term conditions.

The findings are organised into four interlinked categories:

- context of care, network of agents, and interactions;
- continuous adaptations at multiple levels;
- non-linearity, feedback loops, and perceptions of care; and
- clinical empathy and relational continuity as stabilising influences in the system.

### Context of care, network of agents, and interactions

Participants described care for patients with MLTC as a dense and interconnected network of interacting agents, including GPs, hospital specialists, nurses, reception staff, practice managers, patients, paid carers, social workers, and statutory services, as well as non-human agents such as digital systems. They explained how patients with MLTC must operate across multiple organisational boundaries where there was often limited coordination:

*‘My husband has ongoing appointments from five or six different areas of the NHS and social care, and there’s no overall coordination of that centrally. There’s none, you're the one who’s trying to navigate care.’* (Carer, woman, aged 71 years, Midlothian)

This fragmentation was experienced owing to a focus on single conditions, where healthcare professionals often overlooked the cumulative and interacting impacts of MLTC. Although specialisation was recognised as essential to high-quality care, participants felt specialists often lacked an understanding of interacting conditions affecting their care needs. Single-condition-focused pathways proved particularly problematic as this siloed focus undermined person-centred, holistic care and often led to suboptimal experiences of services:

*‘The doctors focus on what a serious problem is and kind of forget about all the others … They forget that you can also get the main problem with many other things.’* (Patient, woman, aged 72 years, Midlothian)

*‘The link between diabetes and mental health issues is not well understood* [by service providers]*, and there is very little in supporting you with both … but two things are feeding into each other, your diabetes gets worse because your mental health is not very good … and then it kind of spirals from there.’* (Patient, man, aged 51 years, Edinburgh)

Participants valued care delivered by generalist professionals with knowledge of their history and sufficient time for meaningful interaction. But time pressures in general practices were seen to limit relational contact:

*‘What you find is GPs say, “We have got only 5–10 minutes”. Before they see the next patient, they sit for 2 minutes trying to run through the screen of what’s wrong with them and their previous problems. By the time* [the] *patient walks in, GPs have forgotten half of what they’ve just read. So they don’t know the patient and they want to go to the next patient as quickly as possible.’* (Patient, man, aged 73 years, Midlothian)

Participants contrasted this with areas where they felt they had been treated well. Cancer services were consistently described as relational, responsive, and well-coordinated, with continuity and clear communication. In contrast, mental health care was characterised by access issues, long waiting times, and limited follow-up, leading many participants to disengage or (for those who could afford it) seek private care:

*‘I am very fortunate. I have a specialist cancer team, and they are brilliant and never dismiss me. I can phone if I'm worried about something, and they will phone me back … I've known them for many years, and they have got to know me as a person.’* (Patient, woman, aged 71 years, Edinburgh)

*‘The mental health support for a stress-related disorder is very difficult to access. I go to a private counsellor and pay because there just didn’t seem to be any counselling services available ... I can’t wait 6 months to talk to somebody as I need help now.’* (Patient, woman, aged 72 years, Edinburgh)

Participants’ contrasting accounts underscored the poor organisation and delivery of care for patients with MLTC that often failed to meet their needs in a timely, holistic, and personalised way.

### Continuous adaptations at multiple levels

Participants recognised that the health and care system was actively adapting to rising demand through structural changes, including the introduction of digital triage systems, new access pathways, and redistribution of clinical tasks among healthcare professionals. However, these system-level adaptations were frequently perceived as misaligned with the complex needs of patients with MLTC. In particular, participants described tensions between standardised, algorithm-driven access models and the relational continuity of care required to manage their multiple interacting conditions:

*‘The doctors are under severe pressure … but this practice alone has 10–11 GPs, and you still can’t get a flaming appointment to see one. You go online and fill in the form and the big problem with that is it’s full of algorithms. If you put one* [answer] *wrong, it kicks you out completely. … I have got to the stage where it will put a question up, and I will go N/A* [not applicable]*, N/A … ALL to get a call and appointment with someone.’* (Patient, man, aged 68 years, Midlothian)

Although digital triage systems were formally introduced to manage demand and streamline access, participants felt these non-human agents were unable to grasp their complex needs. In response, they worked to find ways to circumnavigate the algorithm to reach a provider. Systems therefore generated adaptive patient behaviours that often undermined their original purpose, as individuals modified responses to navigate perceived barriers and secure timely care. Such adaptations highlight the non-linear and emergent nature of agent responses, where patient agency interacts with structural constraints to shape care pathways in unpredictable ways. Some participants adapted to system constraints by working hard to coordinate their own care and/or seeking private health care, particularly where follow-up care was perceived as inadequate or absent. Although this strategy enabled timely access, it raised concerns about equity and widening inequalities:

*‘I think that’s one of the things that will probably come up with all of us. If you are juggling lots of things going on in your body and your mind, you have to be your own advocate, and that is hard work.’* (Patient, woman, aged 75 years, Edinburgh)

*‘The NHS was never a run-for-profit but it’s now getting to the stage where we are getting so underfunded that they* [doctors] *are pushing on you: Well, take out insurance and get it done privately. But what if people can’t afford to pay?’* (Patient, man, aged 73 years, Midlothian)

In the context of sustained resource pressure, informal care emerged as a key adaptive response within the system. Family members increasingly assumed extensive caregiving and coordination roles to try to compensate for gaps in service provision. Participants described how the system had, in turn, adapted to more readily recognise and legitimise carers’ expertise and involvement in care delivery and financing social care:

*‘I found the magic words these days,* [are] *I am a “primary carer”, and that gets you through the door and everybody listens to you.’* (Carer, woman, aged 71 years, Midlothian)

However, the shift in responsibility came without guidance of how to navigate the system, especially following discharge and when seeking social care options:

*‘Social service told us we would have to wait 6 months for care, so we are using a private care firm. We get the basic rate that the council pays and then we top it up ourselves by £3 an hour. It would have been useful if we’d been given a list of private care companies, but we were told go and Google it.’* (Carer, woman, aged 75 years, East Lothian)

An adaptive shift in care often came at a substantial personal cost to carers. Many prioritised the needs of their loved ones over their own health and wellbeing, with limited access to formal carer support services:

*‘The care of my mum and husband is entirely on me. My needs are absolutely ignored and not addressed by social and support services.’* (Carer, woman, aged 56 years, Edinburgh)

### Non-linearity, feedback loops, and perceptions of care

Expectations of care were strongly influenced by prior experiences, with positive encounters fostering trust and engagement and negative interactions having enduring effects on how participants interpreted and navigated future care. Participants consistently described care for MLTC as complex, with care perceptions often shaped by specific interpersonal events:

*‘My husband had a huge operation, and the NHS was fantastic … But all that good was undone because of poor care on the ward afterwards … That led to a long-lasting and devastating effect on us.’* (Carer, woman, aged 75 years, Edinburgh)

Some participants described how suboptimal aspects of care, including delays in medication provision and a lack of responsiveness from professionals, discouraged future engagement with services. In one account, prolonged delays following surgery were experienced not simply as inefficiencies resulting in lapses of care, but as a profound breach of care and trust:

*‘After a big operation, I was left in the ward lying without any liquids. Nurses were trying to get hold of doctors to write a script to get my liquids, but it took hours when I was poor*[ly] *and dehydrated. The operating surgeon came to see me after 5 days, and I said: “You are too late, I am going home”. It was just the most awful care experience, and I lost trust in NHS.’* (Patient, woman, aged 55 years, Edinburgh)

Such experiences illustrate how seemingly discrete or ‘minor’ system delays can have disproportionately negative impacts, including loss of trust and disengagement from care, with one participant noting loudly: *‘I just want to feel cared for’* (patient, man, aged 51 years, Edinburgh). Accounts that undermined empathy in care and personhood were particularly impactful, especially within specialised or acute settings where participants felt highly vulnerable:

*‘When I was admitted to* [hospital]*, a nurse came at 6 am and started to take my pyjama bottoms down. I said: “What are you doing?”,* [she said] *“I'm here to change your pad”. And I went, “I don't wear a pad, and I go to the toilet independently.”* [She responded,] *“Well, how was I to know?”… I thought, if I lost my speech and she treated me like that, I could have actually walloped her.’* (Patient, woman, aged 77 years, East Lothian)

Interactions within the systems produced feedback mechanisms. For instance, repeated unsuccessful attempts to access primary care or escalating concerns frequently resulted in patients seeking unscheduled care:

*‘If you aren’t seeing GPs, what do you do? You go to A&E* [accident and emergency]*.’* (Patient, man, aged 73 years, Midlothian)

Positive feedback mechanisms were also observed, demonstrating how local changes had an impact on perception of care at the practice level:

*‘In our GP practice, the quality of service has really improved under the new manager … it has just transformed the experience of care that you get, because before it felt quite negative ... It’s absolutely about attitude, and you don't feel like you are a problem for the system when you call and see your GP.’* (Patient, woman, aged 71 years, Edinburgh)

Care experiences emerged through dynamic feedback processes, where prior experiences shaped expectations, expectations influenced interactions, and interactions generated outcomes that either reinforced or destabilised trust in the system.

### Clinical empathy and relational continuity as stabilising influences in the system

Across participant discussions, empathetic and relational care were consistently described as stabilising influences within an otherwise fragmented and unpredictable system. Central to such care were acts of compassion, kindness, timeliness, and meaningful information exchanges between agents:

*‘I have a visual impairment. After my surgery, I explained I wouldn’t get the time to learn how to use my stoma. When I was discharged, the nurse talked to me through everything on the letter, in case I couldn’t read it. I needed to be seen by the district nurse team at home, and she phoned and set that up for me. So by the time I left hospital, I felt very secure that things that needed to happen were going to happen, and they had done that for me.’* (Patient, woman, aged 57 years, Edinburgh)

Participants emphasised the importance of relational care and a point of contact who had good interpersonal skills. They noted the importance of interacting with a professional who was familiar with their medical history, who could demonstrate empathy, kindness, and understanding, and who coordinated care across services. Many reflected that sometimes GPs fulfil this role, acting as both gatekeepers and advocates within the system:

*‘Our GP has really gone above and beyond trying to understand how he could potentially help better with my wife’s complex needs. He has been* [an] *exceptionally good communicator and attentive in terms of care and linking her with other specialisms or treatments and following up with us.’* (Carer, man, aged 57 years, Edinburgh)

Where empathy was missing and relational continuity was disrupted, participants at times likened their experience to *‘fighting a system’* rather than receiving joined-up care and good support:

*‘If you could only have one person and they actively listen, discuss and understand your case history, but we see different professionals now and it feels like you are just fighting a system. It is more how it can be good, compassionate, attuned to needs, rather than just a bunch of doctors and procedures that can be done.’* (Patient, man, aged 51 years, Edinburgh)

Patients and carers noted experiencing suboptimal care and breakdowns in informational continuity, especially during care transitions. Post-discharge care, in particular, was characterised by delayed information sharing, limited shared decision making, and inadequate support and follow-up, which intensified feelings of uncertainty and frustration:

*‘Ever since I was discharged, nobody seems to care, know, and communicate what is going on. You never get calls or communication from* [anyone] *anymore … you have to constantly follow up on things yourself.’* (Patient, woman, aged 55 years, Edinburgh)

In response to these gaps, navigator roles emerged as adaptive mechanisms within the system. These roles were not formally designed but were often assumed by kind and caring clinicians to compensate for fragmentations within the broader system:

*‘Having someone qualified, kind, and good in communication would help a lot … A nurse practitioner is ideal, kind of medically literate and able to listen well and empathise when needed. That will make a whole difference to our care.’* (Patient, woman, aged 72 years, Edinburgh)

Clinical empathy and relational continuity were seen as critical in reducing uncertainty, supporting informed decision making, and mitigating the burden of navigating complex fragmented care pathways for people with MLTC and their carers.

## Discussion

### Summary

The findings of this study show how care for patients with MLTC emerged through complex, dynamic, and reciprocal interactions between patients, carers, professionals, and system structures. Single-condition focused care and service adaptations (such as online triage for GP appointments) were frequently perceived as misaligned with their complex needs. Patients with MLTC adapted in turn by modifying their behaviours to achieve their goals, illustrating the emergent, co-evolution of all agents in the system. Care experiences were unpredictable and shaped by individual feedback loops where sometimes seemingly minor encounters had disproportionate effects on care perception, trust, and future engagement. Informal care emerged as an adaptive response to system gaps, with family members increasingly assuming responsibility for coordination, advocacy, and hands-on care, effectively becoming ‘unpaid system agents’. Clinical empathy and relational continuity emerged as key stabilising influences within fragmented systems, enabling shared understanding, meaningful information exchange, and coordination across services, and reducing uncertainty, frustration, and perceptions of treatment burden.

### Strengths and limitations

CAS concepts were used to enable a drawn out nuanced understanding of how care was experienced and perceived, and ways it can be improved to better meet the needs of patients with MLTC and carers. Purposeful recruitment of patients and carers from different HSCPs enabled the inclusion of a wide range of care experiences, thus enhancing the depth and credibility of the analysis. However, recruitment was confined to a single large health board, which may limit transferability to other areas with different organisational configurations or population characteristics. In addition, despite achieving relative balance in age and gender, participants did not include anyone from ethnic and other minority backgrounds. The research team did not have direct access to lists of eligible patients and carers and was therefore unable to purposively sample individuals based on ethnicity. Consequently, the findings may not fully reflect the experiences and perspectives of people from ethnically diverse communities, and future research should prioritise more inclusive recruitment strategies to ensure broader representation.

Furthermore, it is acknowledged that individuals aged <40 years can and do experience MLTC. Their experiences may have distinct system interactions not captured in these data.

### Comparison with existing literature

This study’s findings extend existing evidence showing how patients with MLTC and their carers adapt to compensate for fragmented and poorly coordinated systems, often at the cost of increased patient and carer workload and widening inequities.^19,20^ In line with research on digital health and systems complexity,^21^ algorithm-driven triage and standardised access pathways were experienced as ill-suited to interconnected and complex needs in people with MLTC, generating unintended consequences including exclusion, delayed care, and maladaptive system navigation. These findings firmly reinforce evidence highlighting the importance of empathetic care and continuity as central to high-quality MLTC management.^22,23^ Clinical empathy is recognised as an important component of high-quality care, with previous work demonstrating that perceptions of GP empathy are a strong predictor of patient enablement or capacity to understand and manage their health after consultation.^24^ Building on this evidence, these findings suggest that, in the context of MLTC, clinical empathy may also function as an important adaptive mechanism that helps patients and carers navigate care systems and mitigate some of the negative consequences of fragmentation created by people with MLTC being cared for in systems that are largely single-condition focused. Empathetic care predicts better health outcomes across populations and should, therefore, be actively promoted across healthcare systems, while recognising the challenges of delivering such care in resource-limited settings.^24^ The emergence of findings relating to issues around informal care and navigation roles mirrors previous work in other contexts that demonstrates how system pressures shift coordination and risk onto families^25,26^ and provide inadequate support.^27^

### Implications for research and practice

Good primary care is critical for care of people with MLTC, holding the potential to provide a coordinated gateway to services and a stabilising influence through kind, apathetic, and continuity in care. However, increasing use of digital and algorithmic triage tools conflicts with patients’ expectations of timely access to care from a known GP or other generalist. Patients respond to organisational ‘rules and regulations’ by adapting their behaviour to secure appointments, or bypassing primary care through emergency services, or disengaging altogether. These adaptations, although individually logical, form feedback loops that intensify system strain and widened inequities, particularly for those with limited digital literacy, advocacy skills, or financial resources. Conversely, small positive adaptations and acts of kindness in care can exert disproportionately beneficial effects on care experiences, perceptions, and engagement. Seemingly inconsequential interactions can and do have a disproportionately large impact on patients’ perceptions of care. Clinical empathy and relational continuity function as a stabilising force within otherwise fragmented care systems, facilitating sense making and shared decision making across complex MLTC care pathways.

Better care for patients with MLTC requires more than structural integration or digital innovation because patients’ and carers’ experience of care shapes future behaviour, with outcomes emerging from human relationships and interactions, not linear cause–effect relationships, in a complex adaptive system. Relational continuity must be a core objective, with generalist clinicians providing longitudinal, holistic oversight as stabilising agents who prevent fragmentation and downstream inefficiencies. Furthermore, digital innovations (including large-language model-based chatbots and ‘intelligent agents’) need to be co-designed with patients with MLTC and their carers, augmenting rather than replacing human connection to avoid exclusion and maladaptive responses. Care coordination should be formalised through supported navigator roles embedded in primary or community care, rather than shifting responsibility onto patients and carers. Performance metrics must prioritise what matters to people — empathetic, responsive communication, kind and caring interpersonal interactions, and the reduction of treatment burden. Personalised and kind care needs to be incentivised alongside quality disease care. Finally, supporting carers as key system agents is essential.

Future research should purposively engage and recruit patients with MLTC and their carers in diverse communities to ensure that service redesign is informed by a broader range of cultural experiences, expectations, and structural realities. Without this, attempts at system improvement risk reproducing inequities embedded within existing models of care. Further, research needs to explicitly treat informal carers as co-evolving agents within the care system and explore how caregiving adaptations evolve, sustain, and where crisis points emerge. Understanding which forms of structured, relational, and practical support mitigate inequitable burden will be critical to designing care systems that are not only efficient but equitable, culturally responsive, and sustainable. Finally, the participants in this study had little experience of using artificial intelligence chatbots, but ‘intelligent agents’ built on this technology are likely to be increasingly deployed in health care, for example, to provide health advice or to support triage or appointment booking. Understanding how these technologies influence experience and outcomes of care will be critical.

In conclusion, care for patients with MLTC emerges from dynamic interactions within a complex adaptive system shaped by competing demands for efficiency and person-centredness. These findings show that while system ‘improvements’ often aim to improve efficiency, they can unintentionally destabilise care experiences and shift burden onto patients and their carers. Furthermore, this shift can bring further delays or the avoidance of seeking care altogether, which heightens the risk of avoidable clinical harm. Enabling empathetic and coordinated care through relational continuity, meaningful human connection, and system designs that respect complexity offers a pathway to more sustainable, equitable, and personalised care for patients and their carers.
